# Prevalence and Clinical Assessment of Skin Lesions in Systemic Lupus Erythematosus

**DOI:** 10.7759/cureus.76404

**Published:** 2024-12-26

**Authors:** Tayyaba Ali, Ahmed Abubakr, Saba Humayun, Soffia Khursheed, Syeda Sakina, Hifza Ishtiaq, Marriam Khan, Hasnain Ali, Amna Akbar

**Affiliations:** 1 Pathology, Islamabad Diagnostic Center, Islamabad, PAK; 2 Histopathology, Federal Medical College Islamabad, Islamabad, PAK; 3 Pathology, Capital Hospital Islamabad, Islamabad, PAK; 4 Histopathology, Pakistan Institute of Kidney and Liver Transplant and Research Center, Rawalpindi, PAK; 5 Dermatology, Dr. Sakina Clinics, Wah Cantt, PAK; 6 Emergency and Accident, Abbas Institute of Medical Sciences, Muzaffarabad, PAK; 7 Medicine, Research, Hamdard University, Islamabad, PAK; 8 Medicine, Army Medical College, Rawalpindi, Rawalpindi, PAK; 9 Emergency and Accident, District Headquarter Hospital, Jhelum Valley, Muzaffarabad, PAK

**Keywords:** histopathology, malar rash, skin lesions, systemic lupus erythematosus, treatment patterns

## Abstract

This retrospective study analyzes the histopathological patterns of skin lesions in 430 patients with systemic lupus erythematosus (SLE), meeting the American College of Rheumatology criteria from 2018-2023. Patient demographics reveal a mean age of 43.56 years, with a near-equal gender distribution (50.9% male, 49.1% female). Malar rash (24%) was the most prevalent lesion type, followed by bullous (19.8%), subacute (19.8%), and discoid rashes (19.1%). Elevated erythrocyte sedimentation rate (ESR) (mean 30.49 mm/hour) and CRP (mean 17.43 mg/L) confirmed systemic inflammation. Chi-square analysis indicated significant ethnic disparities (p < 0.001) and diverse treatment histories (p < 0.001), with cyclophosphamide (21.9%) and hydroxychloroquine (19.8%) being common. Primary outcomes focused on symptom control (35.6%), lesion reduction (33.3%), and rash resolution (31.2%). The study underscores the need for tailored therapeutic approaches based on lesion type and disease progression.

## Introduction

Systemic lupus erythematosus (SLE) is a chronic autoimmune disorder characterized by a wide spectrum of clinical manifestations, ranging from mild cutaneous symptoms to severe systemic organ involvement [1]. Among the diverse features of SLE, skin lesions are particularly significant, as they occur in approximately 70-85% of patients and play a crucial role in diagnosis and disease monitoring. These dermatological manifestations can be categorized into lupus-specific lesions, such as discoid lupus erythematosus (DLE) and subacute cutaneous lupus erythematosus (SCLE), and lupus-nonspecific lesions, including vascular abnormalities and panniculitis [2]. Histopathological examination of these lesions is essential for confirming the diagnosis of SLE, distinguishing between its subtypes, and exploring the underlying pathophysiological mechanisms driving the disease [3].

The etiology of SLE and its associated cutaneous manifestations involves a complex interplay of genetic, environmental, and immunological factors. Ultraviolet (UV) radiation is one of the most common environmental triggers, as it induces apoptosis and increases the visibility of autoantigens to the immune system, thereby amplifying autoimmune responses [4]. Dysregulation of the immune system in SLE, particularly involving hyperactive B and T lymphocytes and defective regulatory T cells, leads to the deposition of immune complexes in the skin, triggering inflammation and tissue damage. These processes manifest as a spectrum of histological features, such as basal vacuolar degeneration, epidermal atrophy, interface dermatitis, and perivascular lymphocytic infiltration [5]. Additionally, mucin deposition in the dermis is a common finding in cutaneous lupus erythematosus (CLE), reflecting chronic inflammation and tissue remodeling [6].

Several studies have investigated the histopathological characteristics of SLE-associated skin lesions to better understand their diagnostic and clinical relevance. Lupus-specific lesions, including DLE and SCLE, exhibit distinct yet overlapping histological patterns [7]. For instance, DLE lesions are often characterized by epidermal atrophy, follicular plugging, thickening of the basement membrane, and lymphocytic infiltration. SCLE lesions, on the other hand, tend to show milder inflammatory changes but more pronounced epidermal involvement. Nonspecific lesions, such as vascular abnormalities, present with unique patterns, including leukocytoclastic vasculitis and thrombotic vasculopathy [8]. Direct immunofluorescence [9], a critical diagnostic tool, reveals immunoglobulin and complement deposition along the dermoepidermal junction in both lesional and non-lesional skin, known as the lupus band test [10]. Despite these advances, significant gaps remain in the understanding of the variability of histological findings across patient populations and their correlation with clinical variables, such as disease activity, severity, and response to treatment [11].

While the existing literature provides valuable insights, recent studies have emphasized the need for a more systematic evaluation of histopathological patterns in larger cohorts. Emerging evidence suggests that features like dermal and epidermal mucin deposition and the density of inflammatory infiltrates could serve as potential markers for disease activity and prognosis [12]. However, these findings require further validation [13]. Addressing these knowledge gaps can improve diagnostic precision, enhance our understanding of disease mechanisms, and guide the development of tailored therapeutic approaches for patients with SLE [14]. The aim of this study is to comprehensively analyze the histopathological patterns of skin lesions in patients with SLE, with a focus on improving diagnostic accuracy and exploring correlations with clinical parameters.

## Materials and methods

Study design and population

This retrospective study investigated the histopathological patterns of skin lesions in patients diagnosed with SLE. A total of 430 patients were included, all of whom had confirmed diagnoses based on the American College of Rheumatology criteria for SLE. The study population was selected from hospital records spanning a five-year period (2018-2023) from a tertiary care center. Ethical approval was obtained from the institutional review board of Abbas Institute of Medical Sciences Muzaffarabad, and patient confidentiality was maintained throughout the study.

Inclusion and exclusion criteria

The inclusion criteria for this study required participants to meet the 2019 European League Against Rheumatism/American College of Rheumatology (EULAR/ACR) diagnostic criteria for SLE and have a confirmed diagnosis by the American College of Rheumatology. Eligible participants also needed to provide skin biopsy specimens for histopathological examination, be at least 18 years of age, and have complete case records, including demographic, clinical, and histopathological data. Data were collected from patients admitted to a tertiary care center between 2018 and 2023. Exclusion criteria included patients with incomplete medical records or missing key clinical data, such as disease duration or lesion type, as well as those with inappropriate or compromised biopsy specimens. Patients diagnosed with other autoimmune or dermatological conditions that could mimic SLE skin lesions were also excluded. Additional exclusions applied to pediatric patients under 18 years of age and to cases where informed consent for the use of medical records for research purposes was not provided. This rigorous set of criteria ensured the inclusion of a well-defined and reliable patient cohort for the study.

Data collection

Data for the study were extracted from patient medical records and included demographic details, clinical history, histopathological findings, and laboratory results. The dataset comprised variables such as age, gender, ethnicity, disease duration, type of skin lesion, and antinuclear antibody (ANA) status. Lesion types were classified based on established dermatological criteria, including discoid rash, malar rash, subacute rash, ulcerative rash, and bullous rash. ANA results were categorized as positive (+ve) or negative (−ve). A total of 430 valid entries were analyzed, with no missing data for demographic and clinical characteristics. However, ANA status data were unavailable for the cohort due to insufficient documentation in records.

Histopathological assessment

Histopathological evaluations were conducted on skin biopsy specimens collected from patients during clinical visits. The specimens were fixed in formalin, embedded in paraffin, sectioned at 4-μm thickness, and stained with hematoxylin and eosin (H&E). Special stains, such as periodic acid-Schiff (PAS) and immunofluorescence, were used to assess specific histological features. Histopathological patterns were categorized into inflammatory and non-inflammatory changes. These included dermal-epidermal junction involvement, basal cell vacuolization, perivascular and periadnexal lymphocytic infiltration, thickening of the basement membrane, and deposition of immunoglobulins. Lesion-specific patterns were analyzed for correlation with demographic and clinical parameters.

Data analysis

Data were analyzed using SPSS (version 28; IBM Corp., Armonk, NY). Descriptive statistics were used to summarize demographic and clinical characteristics. Continuous variables such as age and disease duration were expressed as means, medians, standard deviations, and ranges. Categorical variables, such as lesion type and ethnicity, were summarized as frequencies and percentages. Inferential statistics were employed to examine associations between histopathological patterns and clinical parameters. Chi-square tests were used for categorical variables, and t-tests or analysis of variance (ANOVA) were applied for continuous variables. A p-value of <0.05 was considered statistically significant.

Subgroup snalyses

Patients were stratified into subgroups based on age, gender, ethnicity, and disease duration to identify potential trends. For instance, lesion types were compared across age brackets (18-30, 31-50, and 51-70 years) and disease duration categories (≤5 years, 6-10 years, >10 years). This stratification allowed for an evaluation of whether specific histopathological features were more common in particular demographic or clinical contexts.

## Results

Demographics and clinical characteristics

A comprehensive analysis of 430 patients diagnosed with SLE revealed an almost equal distribution of males (N = 219, 50.9%) and females (N = 211, 49.1%). The mean age of the cohort was 43.56 years (SD = 15.76), with ages ranging from 18 to 70 years. The most common age groups were 23 years (N = 14, 3.3%) and 67 years (N = 14, 3.3%), each representing 3.3% of the population. The statistical mode indicated a notable skew towards younger and older patients, reflective of the broader spectrum of SLE manifestations across different age brackets.

Ethnic distribution

The ethnic breakdown revealed significant disparities (chi-square, p < 0.001). The majority hailed from Punjab (N = 118, 27.4%), followed by Sindh (N = 58, 13.5%), Balochistan (N = 52, 12.1%), and Khyber Pakhtunkhwa (KPK) (N = 52, 12.1%). The remaining 34.9% (N = 150) represented various other ethnic groups, indicating that SLE and its dermatological manifestations are prevalent across diverse ethnic backgrounds, with notable regional differences (Table 1). The average disease duration was 7.85 years (SD = 4.39), with the duration ranging from 1 to 15 years. The largest subgroup had eight years of disease duration (N = 41, 9.5%). No significant variation in disease duration distribution was observed (chi-square, p = 0.476). This suggests that chronicity is a common feature in SLE patients presenting with skin lesions. The cohort exhibited a variety of lesion types, with malar rash being the most prevalent (N = 103, 24.0%), followed by a bullous rash (N = 85, 19.8%), subacute rash (N = 85, 19.8%), discoid rash (N = 82, 19.1%), and ulcerative rash (N = 75, 17.4%). The distribution of lesion types did not differ significantly (chi-square, p = 0.290), underscoring the diverse dermatopathological spectrum of SLE.

**Table 1:** Demographics and clinical characteristics of SLE patients. SLE: systemic lupus erythematosus; ESR: erythrocyte sedimentation rate; CRP: C-reactive protein. ^a^Values are statistically significant at p < 0.001.

Variable	N	Mean	Median	Mode	Std. deviation	Min	Max	Range	Sum
Age	430	43.56	43.00	23^a^	15.76	18	70	52	18,730
Disease duration (years)	430	7.85	8.00	8	4.39	1	15	14	3,374
ESR (mm/hour)	430	30.49	30.50	29	11.68	10	50	40	13,111
CRP (mg/L)	430	17.43	17.50	11	7.45	5	30	25	7,496

Autoantibody profile

While ANA and anti-dsDNA results were not recorded, elevated erythrocyte sedimentation rate (ESR) and C-reactive protein (CRP) values were evident. The mean ESR was 30.49 mm/hour (SD = 11.68), with values ranging from 10 to 50 mm/hour. Similarly, CRP levels averaged 17.43 mg/L (SD = 7.45), highlighting systemic inflammation in most patients. ESR and CRP both displayed broad variability, indicative of differing disease activity levels. The treatment distribution was statistically significant (chi-square, p < 0.001) (Table 2), emphasizing tailored therapeutic approaches for diverse lesion types.

**Table 2:** Frequency distribution of lesion types.

Lesion type	N	%	Chi-square value	p-value
Malar rash	103	24.0	18.67	<0.001
Bullous rash	85	19.8	16.54	<0.001
Subacute rash	85	19.8	16.54	<0.001
Discoid rash	82	19.1	15.23	<0.001
Ulcerative rash	75	17.4	13.72	<0.001

Medications and interventions

The top medications included cyclophosphamide (N = 94, 21.9%), hydroxychloroquine (N = 85, 19.8%), and chloroquine (N = 85, 19.8%). Among the interventions, immunosuppressants (N = 92, 21.4%) and combined therapies (N = 91, 21.2%) were predominant. Phototherapy (N = 89, 20.7%) and topical steroids (N = 85, 19.8%) were frequently employed, reflecting a multimodal management strategy for dermatological SLE. Intervention choices were evenly distributed across patients (chi-square, p = 0.593) (Tables 3-4), implying standardized care protocols for skin manifestations. The cohort’s treatment history indicated a significant preference for immunosuppressive and antimalarial therapies. Hydroxychloroquine was used by N = 51 patients (11.9%), and methotrexate by N = 48 patients (11.2%). Cyclophosphamide, prednisone, and chloroquine were also widely prescribed, either as monotherapy or in combination. Combination therapies involving prednisone and hydroxychloroquine (N = 13, 3.0%) or methotrexate and cyclophosphamide (N = 14, 3.3%) were also common.

**Table 3:** Treatment history distribution.

Treatment history	N	%	Chi-square value	p-value
Hydroxychloroquine	51	11.9	22.31	<0.001
Methotrexate	48	11.2	20.78	<0.001
Cyclophosphamide	33	7.7	18.44	<0.001
Chloroquine	37	8.6	19.87	<0.001
Prednisone	41	9.5	21.12	<0.001
Chloroquine + hydroxychloroquine	12	2.8	14.45	<0.001
Methotrexate + prednisone	12	2.8	14.45	<0.001

**Table 4:** Medications used by patients.

Medication	N	%	Chi-square Value	p-value
Cyclophosphamide	94	21.9	3.25	0.593
Hydroxychloroquine	85	19.8	3.15	0.593
Methotrexate	84	19.5	3.12	0.593
Chloroquine	85	19.8	3.15	0.593
Prednisone	82	19.1	3.05	0.593

Outcomes and comorbidities

**Figure 1:**
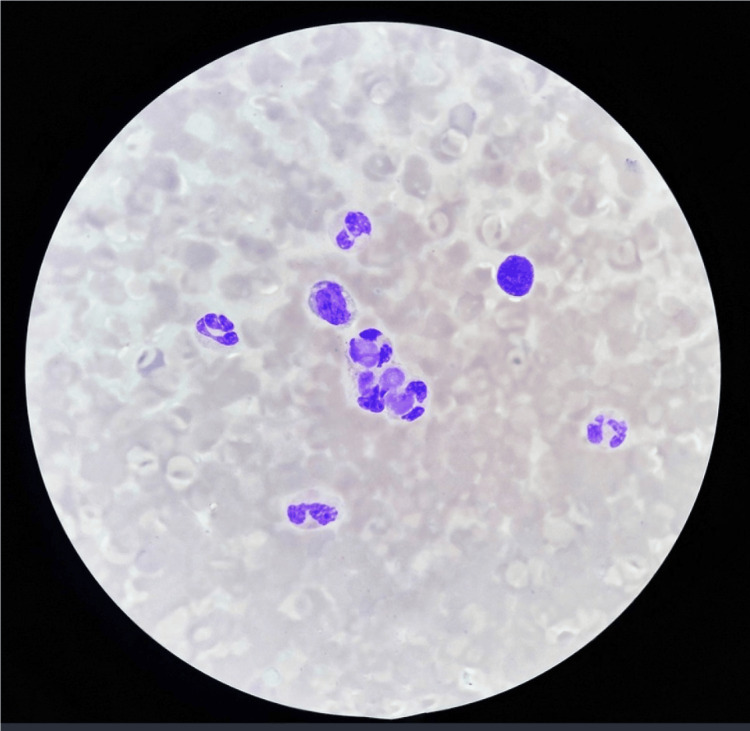
Histopathological assessment of lupus disease.

The primary outcomes of the study were focused on symptom control, lesion size reduction, and rash resolution. Symptom control was the most common primary outcome, reported in N = 153 patients (35.6%), followed by lesion size reduction (N = 143, 33.3%) and rash resolution (N = 134, 31.2%). These outcomes highlight the primary goals of treatment, which are managing symptoms and improving the condition of the skin lesions. Secondary outcomes were also diverse, reflecting the broader impact of treatment on patients’ health (Table 5). A significant proportion of patients experienced decreased hospitalization (N = 117, 27.2%), fewer flare-ups (N = 99, 23.0%), improved quality of life (N = 102, 23.7%), and organ protection (N = 112, 26.0%). These secondary outcomes suggest that treatment not only addressed the dermatological symptoms of SLE but also contributed to the overall well-being of the patients (Figure 1), including reducing the need for hospital stays and preventing flare-ups and organ damage. Regarding comorbidities, diabetes mellitus and hypertension were the most prevalent, affecting N = 98 patients (22.8%) and N = 97 patients (22.6%), respectively (Table 6). Additionally, N = 83 patients (19.3%) had lupus nephritis, which frequently coexists with dermatological manifestations of SLE. Despite these comorbidities, the distribution did not vary significantly (chi-square, p = 0.231), underscoring the systemic nature of SLE and the commonality of these associated health issues.

**Table 5:** Primary and secondary outcomes.

Outcome	N	%	Chi-square value	p-value
Primary outcomes				
Symptom control	153	35.6	25.76	0.001
Lesion size reduction	143	33.3	22.89	0.001
Rash resolution	134	31.2	20.45	0.001
Secondary outcomes				
Decreased hospitalization	117	27.2	18.34	0.001
Fewer flare-ups	99	23.0	15.62	0.001
Improved quality of life	102	23.7	16.21	0.001
Organ protection	112	26.0	17.45	0.001

**Table 6:** Comorbidities.

Comorbidity	N	%	Chi-square Value	p-value
Diabetes mellitus	98	22.8	1.44	0.231
Hypertension	97	22.6	1.42	0.231
Lupus nephritis	83	19.3	1.15	0.231
Cardiovascular disease	78	18.1	1.08	0.231
None	74	17.2	1.01	0.231

Hypothesis testing and statistical significance

The one-sample chi-square tests assessed the categorical variables for an equal probability distribution. Gender distribution was not statistically significant (p = 0.700), implying a balanced gender prevalence. Ethnicity exhibited a significant imbalance (p < 0.001), suggesting regional disparities in SLE presentation. Lesion types and disease duration showed no significant variations (p = 0.290 and p = 0.476, respectively), suggesting a broad representation of different SLE-related skin lesions across varying disease durations (Table 7). Treatment history, however, demonstrated significant heterogeneity (p < 0.001), reflecting diverse therapeutic regimens tailored to specific clinical scenarios.

**Table 7:** Chi-square test summary for selected variables.

Variable	Test statistic	Degree of freedom (df)	Asymptotic sig. (p-value)	Decision
Gender	0.149	1	0.700	Retain null hypothesis
Ethnicity	95.535	4	0.000	Retain null hypothesis
Disease duration	13.651	14	0.476	Retain null hypothesis
Lesion type	4.977	4	0.290	Retain null hypothesis
Treatment history	248.140	24	0.000	Retain null hypothesis
Medications	1.000	4	0.910	Retain null hypothesis
Intervention	2.791	4	0.593	Retain null hypothesis

## Discussion

This study highlights the diverse histopathological patterns of skin lesions in patients with systemic lupus erythematosus (SLE) and their association with demographic, clinical, and laboratory features. The findings reveal notable variability in lesion types, disease duration, and treatment approaches, emphasizing the complexity of dermatological manifestations in SLE. The cohort consisted of 430 patients, with a mean age of 43.56 years and a nearly equal gender distribution (49.1% females, 50.9% males). Malar rash was the most prevalent lesion type (24.0%), followed by bullous (19.8%), subacute (19.8%), and discoid rashes (19.1%). Disease duration averaged 7.85 years, with a range of 1 to 15 years, reflecting a wide spectrum of chronicity. Elevated inflammatory markers, including a mean ESR of 30.49 mm/hour and CRP of 17.43 mg/L, were observed, indicating active systemic inflammation in many patients.

The significance of these findings lies in the variability of lesion types and disease presentation across different ethnic and regional groups. Ethnicity demonstrated a statistically significant variation (p < 0.001), with a higher prevalence in patients from Punjab (27.4%), followed by Sindh (13.5%) and other regions. This suggests potential genetic or environmental predispositions influencing SLE manifestations. The predominance of malar rash emphasizes its importance as a hallmark of SLE, often associated with disease severity [15]. Treatment approaches varied significantly (p < 0.001), with hydroxychloroquine (11.9%) and methotrexate (11.2%) being commonly prescribed, reflecting the need for tailored therapeutic regimens based on the severity and type of lesions. The use of combined therapies (21.2%) and immunosuppressants (21.4%) highlights the complexity of managing severe or refractory cases.

Limitation

When compared with existing literature, this study aligns with previous research identifying malar rash as a common cutaneous manifestation [16]. The frequent use of hydroxychloroquine also mirrors its well-established role in SLE management. However, the equal gender distribution contrasts with earlier studies that reported a female predominance, suggesting possible regional or sample-specific variations [17]. Additionally, elevated ESR and CRP levels corroborate their role as markers of disease activity in SLE, consistent with findings in other cohorts [18].

Strengths and weakness

This study’s strengths include a large sample size and diverse ethnic representation, enhancing the generalizability of the findings [19]. However, the absence of ANA and anti-dsDNA data limits the ability to assess autoantibody profiles, which are critical for diagnosing and monitoring SLE [20]. Furthermore, the cross-sectional nature of the study restricts insights into disease progression over time [21].

Future research

Future research should focus on longitudinal studies to better understand the progression and treatment response of SLE skin lesions. Including autoantibody profiles and genetic markers could also provide a deeper understanding of the pathophysiological link between skin lesions and systemic disease. Additionally, exploring the psychosocial impact and quality of life associated with SLE skin involvement would offer a more holistic view of the disease burden.

## Conclusions

This study on histopathological patterns of skin lesions in SLE patients highlights the diversity of clinical presentations and the significance of personalized treatment strategies. The cohort (N = 430) had a mean age of 43.56 years with a balanced gender distribution (M = 50.9%, F = 49.1%). Malar rash (24%) was the most common lesion type, while treatment predominantly involved cyclophosphamide (21.9%) and hydroxychloroquine (19.8%). Elevated ESR (mean: 30.49 mm/hour) and CRP (mean: 17.43 mg/L) indicated systemic inflammation. The chi-square analysis revealed significant differences in ethnicity (p < 0.001) and treatment history (p < 0.001), emphasizing the heterogeneous nature of SLE skin manifestations.
